# A rare case of palmar congenital nevus with sclerodermoid reaction

**DOI:** 10.1002/ccr3.8241

**Published:** 2024-02-02

**Authors:** Chengbei Bao, Shuyi Shen, Ting Gong, Chao Ji

**Affiliations:** ^1^ Department of Dermatology The First Affiliated Hospital of Fujian Medical University Fuzhou China; ^2^ Key Laboratory of Skin Cancer of Fujian Higher Education Institutions The First Affiliated Hospital, Fujian Medical University Fuzhou China; ^3^ Fujian Dermatology and Venereology Research Institute The First Affiliated Hospital, Fujian Medical University Fuzhou China; ^4^ Central Laboratory The First Affiliated Hospital of Fujian Medical University Fuzhou China

**Keywords:** congenital nevus, desmoplastic, sclerodermoid reaction

## Abstract

Palmar congenital nevus with sclerodermoid reaction has not been reported. It has the potential of deep extension following the fibrous bundle. The utilization of slow Mohs or frozen sections with immunohistochemistry staining was recommended.

## Graphical abstract

A fibrous bundle extending to the superficial fascial layer.

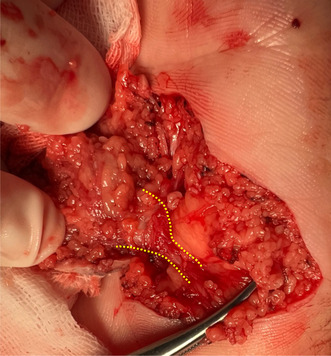

## 1 INTRODUCTION

A congenital melanocytic nevus (CMN) is a skin lesion characterized by benign nevomelanocyte proliferations that present at birth or within the first few weeks.^1^ CMNs are classified based on the maximal diameter into small, medium, and large or giant. Small CMNs are projected to be <1.5 cm in diameter, medium 1.5 to 19.9 cm, and large or giant >20 cm.^1^ An atypical variant of CMN with a hairless presentation, sclerosis, and hypopigmentation has been called “desmoplastic hypopigmented hairless nevus” or “nevus with progressive sclerodermoid reaction.”^2^ Here, we reported a rare case of palmar medium CMN with progressive sclerodermoid reaction which showed deep extended fibrous bundle intraoperatively.

## 2 CASE REPORT

**FIGURE 1:**
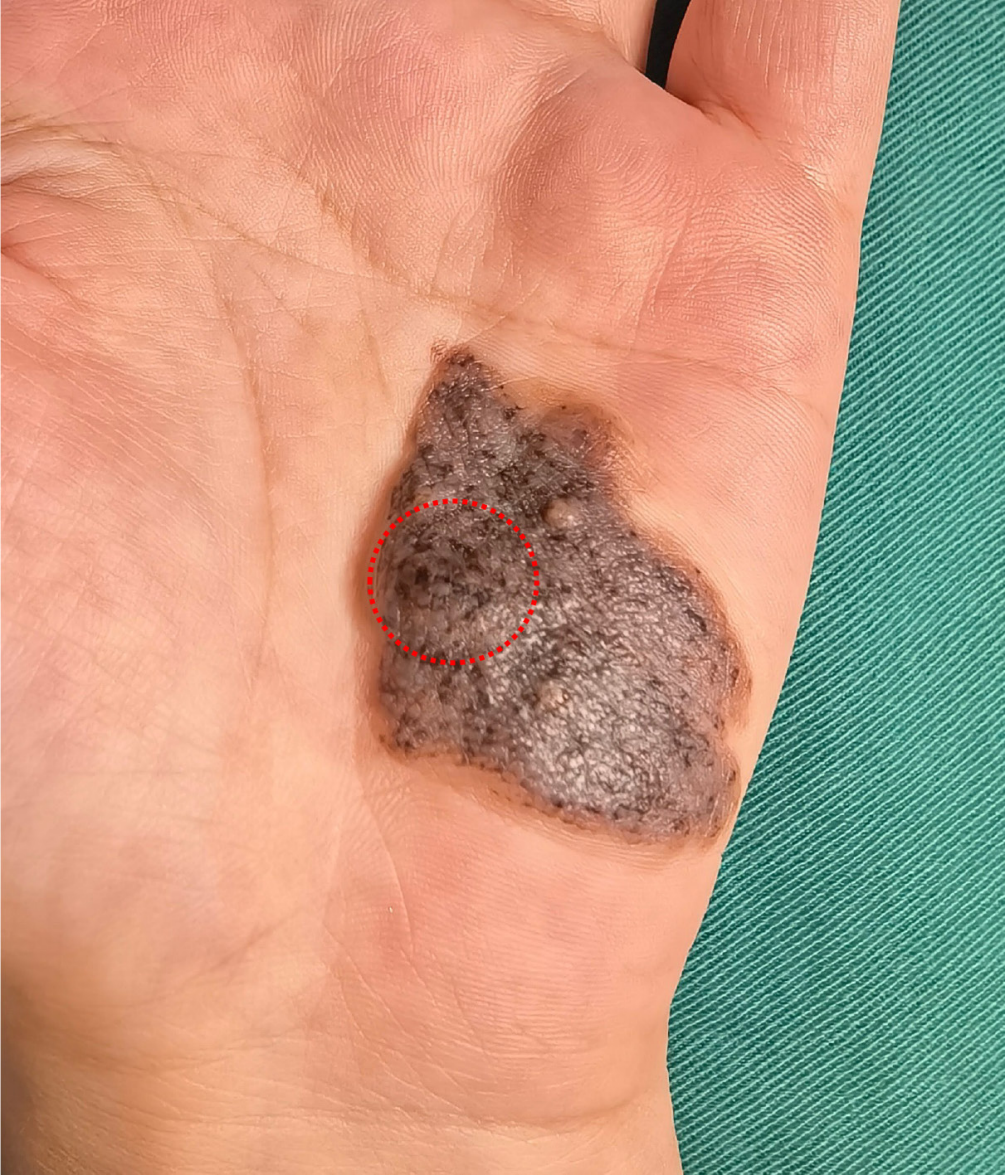
Clinical manifestation of the medium palmar congenital nevus with a focal‐indurated nodule (red dotted circle).

A 30‐year‐old female presented to the Department of Dermatology with a congenital pigmented plaque on the hypothenar eminence. She reported an erythematous patch on the palm at birth with a progressive increase in size and pigmentation. A focal rapidly indurated nodule was developed 1 month prior without markable traumatic history. Physical examination revealed a 3 cm × 3.5 cm, irregular‐bordered, brown‐colored, focal‐indurated patch with a less intensely pigmented perimeter. The non‐tender and indurated nodule was about 1 cm × 1 cm (Figure 1). The dermoscopic examination of the indurated nodule revealed a lattice‐like pattern in a light‐brown background, scattered with some normal‐pigmented areas.(see in Figure S1).

**FIGURE 2:**
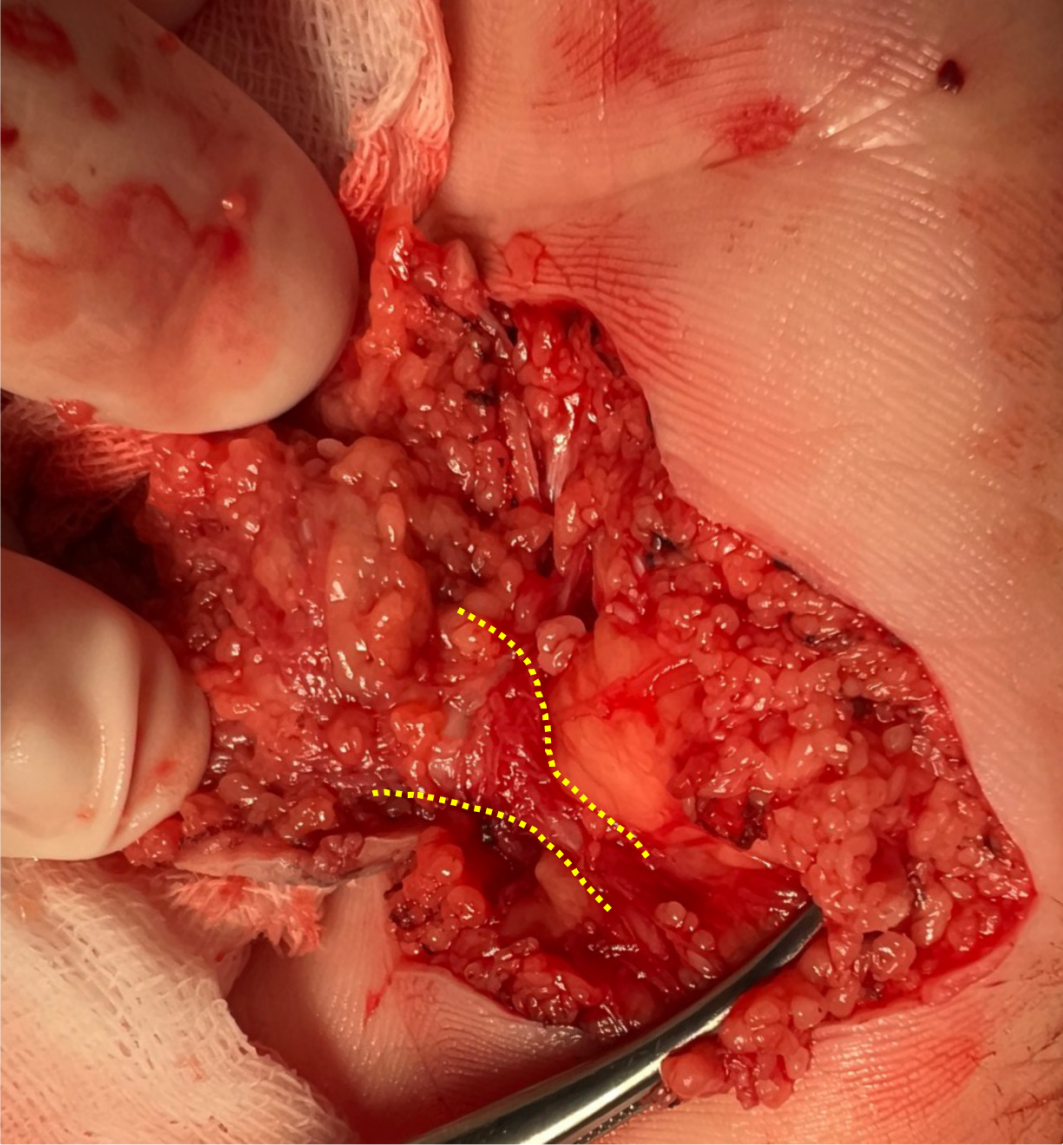
Intraoperative image showing a fibrous bundle (yellow dotted line) connected to the nodule with an extension into the superficial fascial layer.

**FIGURE 3:**
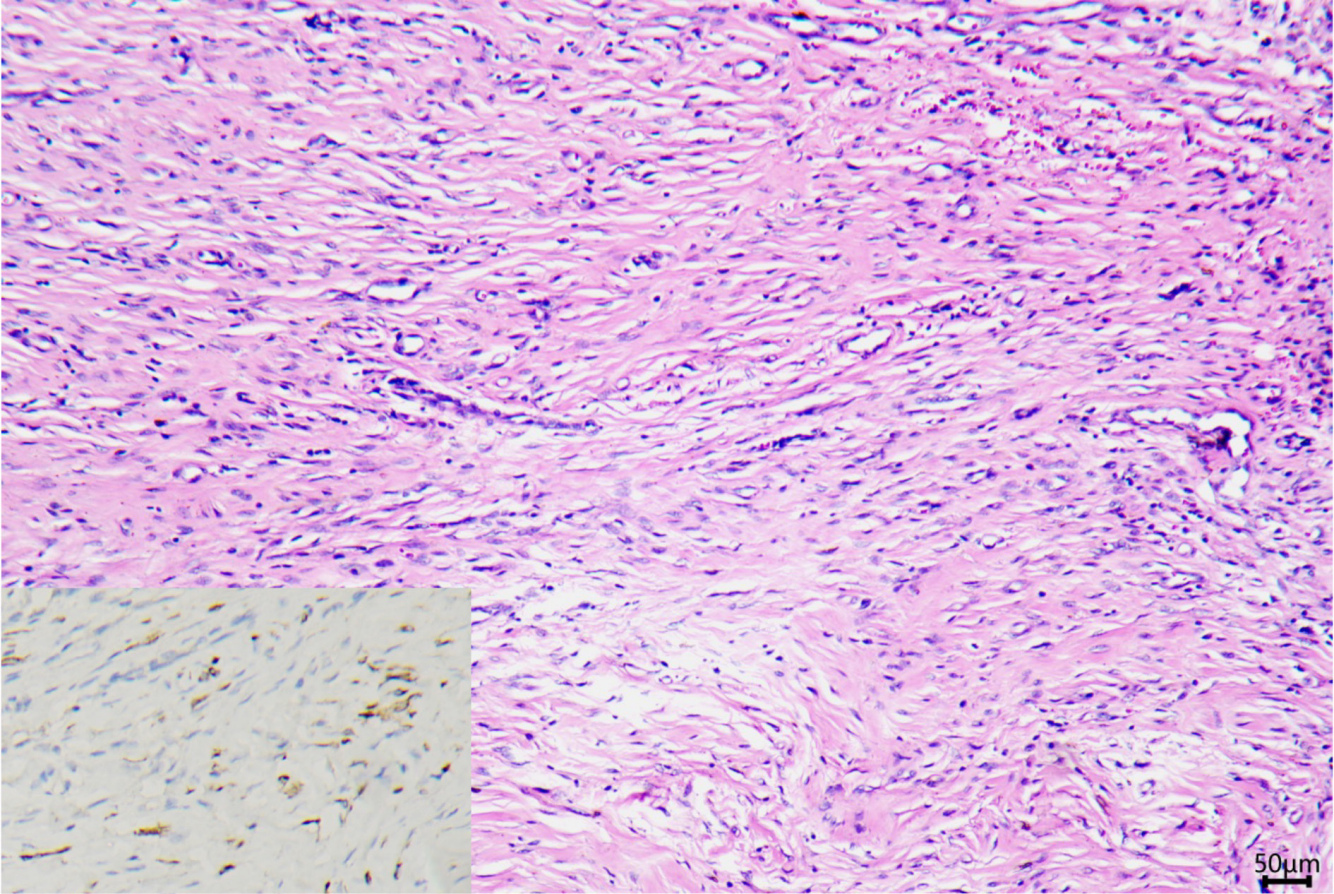
Prominent collagen bundles and spindle cell population in the basal incision edge (H&E 100×) and immunohistochemistry staining of Melan‐A highlighted the sparse, spindle‐shaped nevus cell (inbox).

Complete excision with frozen sections examination was performed based on the patient's requirement and the possibility of malignant transformation. The whole lesion was incised with a 1 mm margin. Intraoperatively, a fibrous bundle connected to the nodule with an extension into the superficial fascial layer was detected (Figure 2). We isolated the fibrous bundle at 5 mm from the nodule. Frozen section examination reported negative results of atypical melanocytic nevus cells in the indurated nodule, with a hypercellular fibrous bundle at the basal edge. The H&E sections revealed melanocytic nests in the superficial dermis and sheets in the reticular dermis, which is consistent with congenital nevus. The immunohistochemical (IHC) staining results showed BRAF (+), Cyclin D1 (−), HMB45 (−), Ki67 (1%+), MITF (+), Melan‐A (+). P53(−), PNL2 (−), S100 (+), and SOX10 (+). The basal incision edge, a hypercellular collagenous bundle, revealed the spindle cell population with sparse spindle‐shaped nevus cells (Figure 3), indicating the potential for nevus cell extension following the fibrous bundle. The immunohistochemistry staining of Melan‐A for the second excision specimen at another 5 mm of the fibrous bundle reported a negative result. Palmar medium CMN with sclerodermoid reaction was diagnosed based on the congenital history and prominent histologic desmoplasia. The defect was treated in the second stage with a free skin graft and vacuum sealing therapy. The skin graft survived, and the patient was regularly followed up.

## 3 DISCUSSION

A previous study summarizes the features of desmoplastic hypopigmented hairless nevus, which revealed a preference for large congenital nevi in hairy skin, such as the trunk, face, and extremities, with a benign evolution in all described cases.^2^ The sclerodermoid reaction may result from the differentiation of melanocytes into fibroblasts or the interactions between the tumor extracellular matrix which led to the stimulation of collagen synthesis by fibroblasts.^2^ The hairless presentation may be attributed to the hypotrophy of hair follicles that trapped by proliferative connective tissue.^2^ To our knowledge, the case described herein is the first exclusively located on the palm. As volar palm cannot meet the “hairless” feature, we prefer to diagnose the disease as “palmar CMN with the sclerodermoid reaction.”

The primary differential diagnosis was acral melanoma(AM), as malignant melanoma transformation could be a complication of CMNs. A recent study reported an incidence of 1.55% (8/514) of children and adolescents suffering from CMN‐associated melanoma.^3^ Clinically, AM manifests with irregular pigmentation, nodules, and ulceration, which is partly consistent with our case. Although AM accounts for only 2%–3% of all melanoma cases, it is the most common subtype in Asians, which accounts for approximately 41.8% of melanoma in China.^4^

Treatment modalities include surgery and observation without active intervention.^5^ Excision has been recommended as the first‐line treatment in which treatment is indicated, with high satisfaction rates when CMN are<20 cm in size.^5^ Although previous studies reported a benign course, we recommend excision of acral CMN with focal sclerodermoid reaction.^2^ It is based on the susceptibility of the palm to friction, the tendency for deep extension of the nevus cell along the fibrous bundle, and the significant prevalence of AM in Asian populations.^4^ As our case showed that nevus cells in fibrous bundles interspersed with spindle fibroblasts, we recommended performing slow Mohs or frozen sections with IHC staining in similar cases to determine the residual nevus cells in the fibrous bundles.

## 4 CONCLUSION

In cases of palmar CMN exhibiting rapid focal induration, it is crucial to consider the possibility of CMN with a sclerodermoid reaction. We highly recommend the utilization of slow Mohs or frozen sections with IHC staining to minimize the risk of recurrence.

## AUTHOR CONTRIBUTIONS

**Chengbei Bao:** Resources; writing – original draft. **Shuyi Shen:** Resources. **Ting Gong:** Writing – review and editing. **Chao Ji:** Conceptualization.

## FUNDING INFORMATION

None to declare.

## CONFLICT OF INTEREST STATEMENT

None to declare.

## ETHICS STATEMENT

The patients in this manuscript have given written informed consent to the publication of case details.

## Supporting information


**Supplementary Figure 1.** The dermoscopic result of the indurated nodule which revealed a lattice‐like pattern in a light‐brown background, scattered with some normal‐pigmented areas.

## Data Availability

The data that support the findings of this study are available on request from the corresponding author. The data are not publicly available due to privacy or ethical restrictions.
